# Effect of Sodium Chloride Concentrations on Processing Characteristics and Quality of Mianpi Made Using Different Wheat Flour–Starch Levels

**DOI:** 10.3390/foods14173127

**Published:** 2025-09-06

**Authors:** Yang Lu, Luo Tang, Shuying Li, Peiling Liu, Ting Chen, Fayin Ye

**Affiliations:** 1 College of Food Science, Southwest University, Chongqing 400715, China; ly1271761473@163.com (Y.L.); t18582181862@163.com (L.T.); 15922947901@163.com (S.L.); 15283548452@163.com (P.L.); 2 Chongqing Fengdu Center for Disease Control and Prevention, Chongqing 408200, China; 3 Chongqing Jiulongpo Center for Disease Control and Prevention, Chongqing 400039, China

**Keywords:** Sodium chloride addition, wheat flour–starch mix, wheat starch gel food, physicochemical properties, structural characteristics of starch

## Abstract

Sodium chloride (NaCl) was essential for making mianpi, a traditional Chinese wheat starch gel food. The production process included wheat flour/starch slurry preparation, steaming, cooling, and cutting. This study investigated how NaCl affected both the slurry’s properties and the quality of mianpi using three formulations: wheat flour (F100), a 50:50 (*w*/*w*) wheat flour–starch mix (F50), and wheat starch (F0). Our findings demonstrated that NaCl significantly altered the slurry rheology, pasting behavior, texture, and starch ordered structures. It notably reduced the slurry apparent viscosity, while it showed a divergent effect on its pasting properties. Regarding product quality, NaCl induced a measurable alteration in L*, a*, and b* values of mianpi, though visually imperceptible. F100 mianpi maintained texture except for when adding 2% NaCl, which reduced hardness. NaCl increased tensile strength (excluding F0). However, it caused irregular texture changes in F50 and F0 mianpi. Furthermore, NaCl modulated viscoelastic properties of mianpi products, as evidenced by reductions in storage and loss modulus. FT-IR showed NaCl disrupted starch short-range order in F100/F0 but improved it in F50, though Raman spectroscopy (480 cm^−1^) did not detect this shift. Gluten protein secondary structure remained unaffected across all formulations. These results guide NaCl–starch–flour formulations in starch-gel-based foods.

## 1. Introduction

As a widely utilized ingredient in the food industry, NaCl primarily functions to enhance sensory attributes such as flavor and palatability, while it also contributes to the modification of textural properties [1]. For the manufacture of wheat-flour-based food products, NaCl is frequently incorporated as a fundamental ingredient to promote product quality, particularly in terms of texture and structural integrity [2]. As is known to all, NaCl plays a critical role in bread making [3]. NaCl helps to form and strengthen the wheat gluten network, stabilize yeast fermentation, and even enhance the product flavor [4,5]. Carcea et al. [6] confirmed that a proper amount of NaCl (1.5%, *w*/*w*) was helpful to improve the technological properties of medium breadmaking quality flours. Results also indicated that NaCl was responsible for the color of bread crust, texture of crumb, and the specific volume of bread [4]. Additionally, NaCl showed an impact on the processing properties of wheat flour and wheat-based noodle quality. Fan et al. [7] found that the stability time and the resistance to extension ratio of wheat flour was increased by NaCl addition; meanwhile, the gluten network of noodles became denser with increasing NaCl concentration. Wang et al. [8] found that the addition of NaCl improved the extensibility of dough, and they concluded that dough with the addition of an appropriate amount of NaCl (at level of 3–5%, *w*/*w*) was essential for making traditional Chinese hand-stretched dried noodles.

Mianpi, a traditional Chinese food, is typically made from wheat flour with a chewy, thick, and translucent texture and appearance. Mianpi is similar in shape to Chinese wheat noodles. However, its making process is quite different. Mianpi can be broadly categorized into two types based on its production process: fermented and non-fermented. Fermented mianpi is prepared by firstly mixing wheat flour with water to form a dough, washing the dough to separate the starch-rich slurry, and then subjecting the slurry to short-term natural fermentation before cooking [9]. The conventional preparation process of non-fermented mianpi typically includes wheat flour slurry mixing, steaming, cooling, and cutting. As such, the structural development of the final product primarily relies on the gelatinization and gelation of wheat starch during thermal processing, which leads to the formation of a three-dimensional gel network that provides structural integrity. Therefore, mianpi is a type of starch-based gel food. In order to achieve a denser, harder, slippery texture, the component of wheat flour was adjusted prior to mianpi making by adding some amount of wheat starch or removing the gluten in a gluten wash-out process [9]. Similar to the processing of noodles [7,10], NaCl is essential in mianpi making. In addition to enhancing its sensory properties, NaCl is added for its impact on the processing handling of the slurries and the final texture characteristics of mianpi.

**Figure 1:**
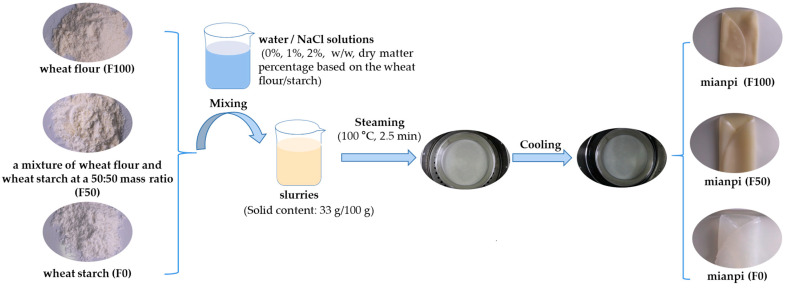
The manufacturing process of mianpi and the preparation scheme for mianpi samples with varying concentrations of NaCl and ratios of wheat flour to wheat starch in the slurry.

Several studies [11,12,13] have confirmed that NaCl could modify the techno-functional properties of wheat flour constituents (e.g., protein and starch). Moreover, NaCl shows impact on the physicochemical properties of various extracted starches [14,15]. Zheng et al. [16] found that a moderate amount of NaCl addition (150 mM) is helpful for the 3D printing performance of wheat starch and the quality of 3D-printed wheat starch gel. However, the effects of NaCl on the qualities of mianpi remain insufficiently elucidated. Therefore, this study was performed to analyze the effect of NaCl addition on the processing characteristics of wheat flour slurries and qualities of mianpi made thereof. According to Figure 1, the slurries are made from (i) wheat flour (F100), (ii) a mixture of wheat flour and wheat starch at a 50:50 mass ratio (F50), and (iii) wheat starch (F0). These three formulations represent extremely distinct recipes: F100 as a gluten-rich system, F0 as a starch-dominated system, and F50 as an intermediate mix. Comparing these systems allows us to clarify the differential roles of starch and gluten in determining the functional impact of NaCl and to highlight how compositional variation influences product properties. We aim to clarify the changes in techno-functional properties of mianpi materials induced by NaCl addition, and the findings of this study might provide insights for developing recipes for the making of mianpi and improving mianpi quality in an industrial scale.

## 2. Materials and Methods

### 2.1. Materials

Wheat flour was obtained from COFCO International (Beijing, China), with moisture 13.39%, starch 69.57%, protein 12.62%, fat 0.99%, ash 0.40% in the flour. Wheat starch was purchased from Baoji Yuansheng Industrial Co., Ltd. (Baoji, Shaanxi, China), with moisture 12.47%, starch 81.80%, protein 0.8%, fat 0.57%, and ash 0.22%. Food-grade sodium chloride (NaCl ≥ 99.1%) was provided by China National Salt Industry Corporation (Chengdu, Sichuan, China). All the chemical reagents used were of analytical grade.

### 2.2. Preparation of Mianpi Slurries

Mianpi slurries, namely, the aqueous dispersions of wheat flour (designated as F100), wheat flour–starch blend (50:50 by weight, dry matter; designated as F50), or wheat starch (designated as F0), were prepared by mixing with water or NaCl solutions in a blender (YM-B80, Yanmai Food Machinery Co., Ltd., Shanghai, China). The obtained slurries had a solid content of 33%. The amount of NaCl added in the slurries was set as 0%, 1%, and 2%.

### 2.3. Flow Behavior Analysis

A rheometer (DHR-2, TA Instruments, New Castle, DE, USA) was used in determining the flow behavior of the slurries F100 and F50. F0 was not measured due to the high tendency to settle. Slurry samples were placed on the plate of the rheometer (equipped with a parallel-plate geometry, 40 mm diameter, 1000 µm gap) and maintained at 25 °C for 3 min. The flow behavior was analyzed at 25 °C, with temperature control of ±0.1 °C provided by the Peltier plate. Strain-controlled shear data (shear stress and apparent viscosity) were automatically recorded across a shear rate range of 0.1–150 s^−1^. The flow parameters of the slurries were fitted with the power law model (Equation (1)):

(1) $$\tau =K\cdot \gamma^{n}$$

where *τ* is the shear stress (Pa), *K* is the consistency index (Pa·s*ⁿ*), *γ* is the shear rate (s^−1^), and *n* is the flow behavior index (dimensionless).

### 2.4. Rapid Visco Analyzer (RVA)

The pasting properties of the flours (F100, F50, F0) with different amounts of NaCl (0%, 1%, 2%, 4.46%, and 8.92%, *w*/*w*, percent dry matter in unit mass) were determined using a Rapid Visco Analyzer (RVA 4800, Perten Instruments AB, Hägersten, Stockholm, Sweden) according to the Chinese National Standard GB/T 24853-2010 [17] (Wheat, Rye, and Their Flours and Starches—Determination of Pasting Properties by Rapid Viscometer Method). Namely, a 3.50 g portion of the sample (corrected to 14% moisture basis) was accurately weighed and mixed with 25.0 g of distilled water. The mixture was immediately loaded into the RVA sample canister for testing. The Standard 1 program was used with the following settings: the paddle speed was set at 960 rpm for the initial 10 s, then maintained at 160 rpm for the remainder of the test. The temperature program consisted of holding at 50 °C for 1 min, heating to 95 °C over 3.7 min, holding at 95 °C for 2.5 min, cooling back to 50 °C over 3.8 min, and finally holding at 50 °C for 2 min [15]. The resulting pasting curves and pasting parameters were recorded for each sample.

### 2.5. Preparation of Mianpi

For each sample, 70 g of the slurry obtained in Section 2.2 was evenly distributed in an iron flat pan (*ϕ* = 19 cm). The slurry-containing iron flat pan was then transferred to a steaming chamber (pre-heated with boiling water in advance) on an induction cooker (C22-RT22E01, Guangdong Midea Consumer Electric Manufacturing Co., Ltd., Foshan, Guangdong, China) and steamed at 100 °C (1 atm) for 2.5 min to obtain a cooked paste. After 2.5 min steaming, the iron flat pan was taken out immediately and cooled to room temperature within 5 min by floating it in a cooling water system [18]. The cooked paste transformed into mianpi during the cooling period. The as-prepared samples were wrapped with food-grade polyethylene film for further analysis.

### 2.6. Color Measurement of Mianpi

The color of the samples was measured using a colorimeter (UltraScan PRO, Hunter Associates Laboratory, Inc., Reston, VA, USA) based on the CIE L* a* b* color space system. Prior to measurement, the instrument was calibrated using a standard black and white reference tile. Color values were recorded at six different positions on each sample. The total color difference (Δ*E*) was calculated using Equation (2):

(2) $$\Delta E=\sqrt{{\left(L^{*}-{L_{0}}^{*}\right)}^{2}+{\left(a^{*}-{a_{0}}^{*}\right)}^{2}{+\left(b^{*}-{b_{0}}^{*}\right)}^{2}}$$

In the equations, L*, lightness, a*, level of redness or greenness, and b*, level of yellowness or blueness, where ${L_{0}}^{*}$, ${a_{0}}^{*}$, and ${b_{0}}^{*}$ were the color parameters for the control samples. In this study, the mianpi without adding NaCl was used as the control samples for calculating the Δ*E*.

### 2.7. Texture Profile Analysis (TPA)

TPA of the mianpi samples was performed on a texture analyzer (TA. XT Plus, Stable Micro Systems, Godalming, Surrey, UK). Prior to the testing, the samples were cut into strips (about 5 cm in length and 1 cm in width) and two strips were overlapped (final thickness approximately 2.4 mm) and placed on the test platform. A P/36R cylindrical probe was used for compression testing and the parameters were as follows: pre-test speed, 2 mm/s; test speed, 1 mm/s; post-test speed, 1 mm/s; compression strain, 50%; interval between two compressions, 5 s; trigger force, 5 g [7].

### 2.8. Tensile Properties of Mianpi

Prior to the testing, the mianpi samples were cut into strips of 5 cm (length) × 1 cm (width). The texture analyzer (TA. XT Plus, Stable Micro Systems, Godalming, Surrey, UK) equipped with a tensile testing device (A/KIE tensile fixture) was used for the tensile analysis [14]. The test parameters were set as follows: pre-test speed, 5 mm/s; test speed, 1 mm/s; post-test speed, 5 mm/s; trigger force, 5 g; and stretching distance, 50.0 mm [19].

### 2.9. Frequency Sweep Test of Mianpi

The frequency sweep analysis of the samples was conducted using a rheometer (DHR-2, TA Instruments, New Castle, DE, USA) in oscillation mode. The rheometer was equipped with a parallel-plate geometry (40 mm diameter, 1000 µm gap). The mianpi samples were prepared as described in Section 2.5 and were cut into circular discs (50 mm diameter) and then placed at the center of the rheometer platform. The edges were sealed with silicone oil to prevent moisture loss. Before testing, the sample was equilibrated at 25 °C for 1 min to eliminate internal stress [16]. A strain sweep was first performed at a constant angular frequency of 10 rad/s over a strain range of 0.01–100% to determine the linear viscoelastic region (LVR). Subsequently, a frequency sweep was carried out within the LVR at a constant strain of 0.1% and temperature of 25 °C, over an angular frequency range of 0.1–100 rad/s. The storage modulus (*G′*), loss modulus (*G*″), and loss factor (tan *δ*) were recorded.

### 2.10. Raman Spectroscopic Analysis of Mianpi

Raman spectra of the mianpi samples were acquired using a confocal Raman spectrometer (inVia™, Renishaw plc, Wotton-under-Edge, Gloucestershire, UK) with an excitation wavelength of 785 nm. The scanning range was 100–3400 cm^−1^, with an excitation power of 8 mW, an integration time of 6 s, and an accumulation of 10 scans. The spectrometer was equipped with a 600 lines/mm grating and a pinhole aperture of 1.3 µm [20]. Ambient air spectra were used as the background reference. The full width at half maximum (FWHM) of the characteristic peak at 480 cm^−1^ was calculated using Origin 2022 software (OriginLab Corporation, Northampton, MA, USA).

### 2.11. Fourier Transform Infrared (FTIR) Spectroscopic Analysis of Mianpi

Prior to FTIR analysis, the mianpi samples were freeze-dried with a vacuum freeze-dryer (SCIENTZ-10ND, Ningbo SCIENTZ Freeze-Drying Equipment Co., Ltd., Ningbo, Zhejiang, China). The freeze-dried samples were ground with a grinder (BJ-150, Yongkang Hongtaiyang Electromechanical Co., Ltd., Jinhua, Zhejiang, China) and then passed through a 100-mesh sieve. FTIR spectra were recorded using a Fourier transform infrared spectrometer with an attenuated total reflectance (ATR) accessory (Spectrum100, Perkin-Elmer, Waltham, MA, USA) in the range of 600–4000 cm^−1^, with a resolution of 4 cm^−1^ and 32 scans. Air was used to determine the background spectra. The spectra were plotted with wavenumber on the *x*-axis and absorbance on the *y*-axis. Deconvolution of the bands between 800 and 1200 cm^−1^ was performed using OMNIC software (Version 9.2, Thermo Fisher Scientific, Madison, WI, USA). The FWHM was set to 40 cm^−1^, with an enhancement factor of 1.9. The absorbance ratios (*R*_1047/1022_ and *R*_1022/995_) of the samples at bands 1047, 1022, and 995 cm^−1^ were calculated to evaluate the short-range ordered structure of starch in the samples. The amide I region (1600–1700 cm^−1^) was analyzed using PeakFit software (v4.12, SeaSolve Software Inc., San Jose, CA, USA) [21]. Baseline correction and second-derivative fitting were applied to quantify the secondary structure of protein components.

### 2.12. Statistical Analysis

The experimental data were expressed as the mean ± standard deviation. The figures and charts were drawn by using Origin 2022 (OriginLab Corporation, Northampton, MA, USA). The experimental data were processed by SPSS 26.0 (IBM Corporation, Armonk, New York, NY, USA), and Duncan’s multiple range test was used to analyze significant difference levels (*p* < 0.05) using one-way analysis of variance (ANOVA).

## 3. Results and Discussion

### 3.1. Effects of NaCl on Flow Properties of the Slurries

**Figure 2:**
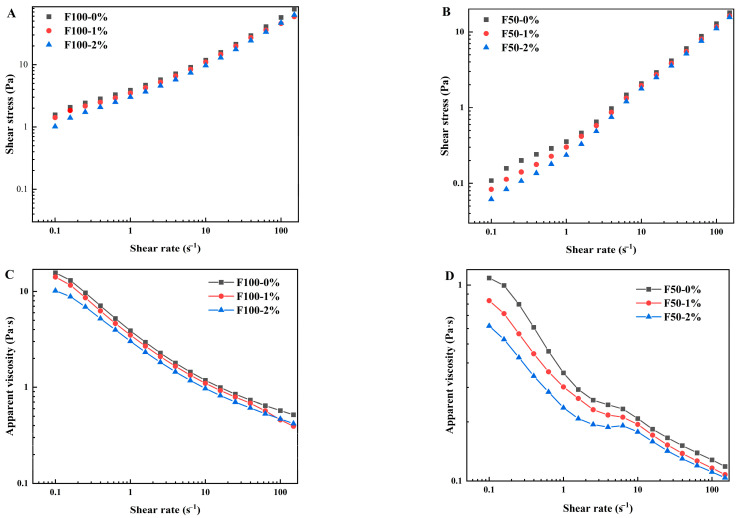
Shear stress (**A**,**B**) and apparent viscosity (**C**,**D**) versus shear rate curves of the slurries prepared from wheat flour (F100) and wheat flour–starch mix (F50) with varying NaCl concentrations (0%, 1%, 2%).

In industrial-scale mianpi production, the slurry is typically transported via pipelines to a steaming chamber for cooking. Under these conditions, the slurry’s flow properties become critical for both uniform spreading during processing and efficient pipeline transport. Steady shear measurements can effectively characterize these flow behaviors. The results (Figure 2) showed that F100 and F50 slurries displayed typical shear-thinning behavior, in which the apparent viscosity of both slurries decreased with increasing shear rate. F0 slurry failed to produce a good flow curve, i.e., a plot of shear stress versus shear rate, because the slurry was liquid-like and when loaded onto the rheometer the wheat starch in the slurry easily settled on the bottom. F50 slurries had a lower apparent viscosity than F100 over the shear rate range (0.1–150 s^−1^). This phenomenon primarily resulted from the higher content of gluten proteins and other hydrophilic substances in F100 compared to F50. These components bound water molecules, conferring greater viscosity to the slurry. When wheat flour was 50% replaced by wheat starch, the gluten proteins in the slurry were diluted, leading to an increase in available free water in the slurry. This free water acted as a lubricant for particles, enhancing flowability and reducing viscosity. Moreover, unlike F100 slurries, F50 slurries showed a Newtonian-like plateau in the shear rate range of 1–10 s^−1^. Wang et al. [15] also reported that the starch dough (i.e., a dispersion of starch granules in a continuous matrix of starch paste) showed a Newtonian-like plateau at shear rates of 1–10 s^−1^. This phenomenon was probably the result of the combined action of two effects, i.e., the shear-thinning trend of the aqueous phase (mainly containing solutes, such as salt ions and soluble proteins) and a shear-thickening effect of the solid phase (consisting of starch granules and aggregated gluten proteins) [22]. In F100 slurry, gluten proteins were interconnected through disulfide bonds and extensive physical entanglements, forming a polymer-like network that was resistant to deformation [23]. Under applied shear, non-covalent interactions and entanglements were partially disrupted, leading to a progressive decrease in apparent viscosity with increasing shear rate. By contrast, in the F50 slurry, the added starch granules changed the flow behavior response. Under shear, starch granules tended to orient along the flow direction [15], thereby reducing hydrodynamic resistance. Meanwhile, gluten entanglements and weaker non-covalent contacts still affected the rheological characteristics of the slurry, but their effect was moderated by the diluted protein fraction and particle–protein interactions. Thus, the shear-thinning observed in both systems arose from network disruption and reorganization in gluten, while in F50 particle alignment additionally facilitated the flow. The results (Figure 2) also showed that the added NaCl decreased the slurry’s apparent viscosity. To quantitatively analyze the rheological response upon the addition of NaCl, the power-law model was applied for fitting the flow curves. As shown in Table 1, the correlation coefficients (*R*^2^) for all samples ranged from 0.997 to 1.000, indicating that the power-law model fitted the rheological behavior of all samples. The flow behavior index (*n*) for all samples was less than 1, confirming the pseudoplastic flow behavior [24]. The *n* values for F50 slurries were consistently above 0.8, indicating a relatively weak shear-thinning effect. The consistency coefficient (*K*) characterized the consistency or thickness of a fluid. As the wheat starch substitution increased from 0% to 50%, the *K* value decreased from 2.27 Pa·s*^n^* to 0.29 Pa·s*^n^*. Similarly, Seleh [25] reported that the batter formulated with 100% wheat flour exhibited an *n* of 1.207 and a *K* of 5.00 mPa·s*^n^*—both significantly higher than those of samples containing varying proportions of starch or non-wheat flour/egg levels. The added NaCl showed an insignificant effect on *K* and *n* values of F50 slurry. However, NaCl, at concentrations of 1% and 2% (*w*/*w*), exerted opposite effects on the consistency coefficient (*K*) and flow behavior index (*n*) of the F100 slurry. Results indicated that wheat proteins played the key role, and 1% (*w*/*w*) NaCl increased the solubility of wheat proteins so that the *K* value was significantly increased (*p* < 0.05) and 2% (*w*/*w*) NaCl decreased protein hydration to achieve an opposite trend. Xue and Ngadi [26] found that 2.5% (*w*/*w*) NaCl significantly lowered the viscosity of cereal flour batter.

### 3.2. Effects of NaCl on Pasting Properties of the Slurries

**Figure 3:**
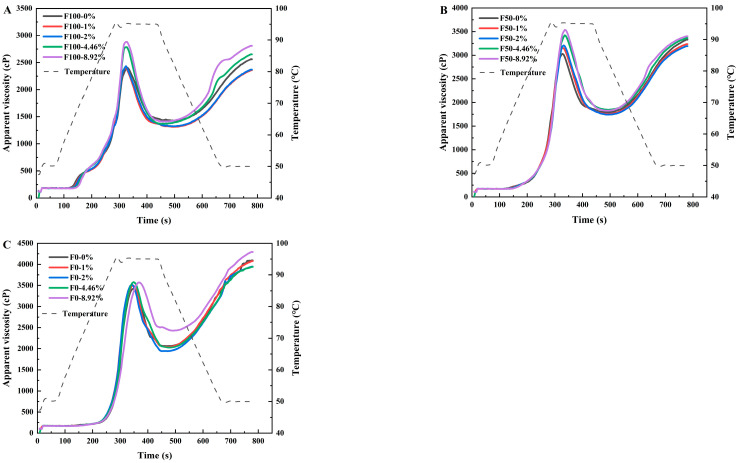
Pasting profiles of wheat flour (**A**), wheat flour–starch mix (**B**), and wheat starch (**C**) with different levels of NaCl.

The pasting properties measured by rapid visco-analysis (RVA) represent the viscosity changes in the wheat flour/starch suspensions throughout the heating and cooling cycle, serving as an indicator of the molecular dynamics within starch granules. Starch pasting is a pre-condition for starch gel formation which is essential in the process of mianpi making. The viscosity parameters are related to processing characteristics of the wheat flour/starch slurries and qualities of mianpi products. Figure 3A–C show the pasting profiles of wheat flour (F100), mixture of wheat flour and wheat starch (F50), and wheat starch (F0) with different addition amounts of NaCl, respectively. Upon the programmed rapid heating, the sample viscosities grew and reached a peak quickly and showed a steep profile. By holding at the maximum temperature (95 °C), a drop in viscosity occurred in all samples due to the disruption of swollen starch granules. The viscosity steadily grew in the cooling stage. At the lower NaCl concentrations (1% and 2%), NaCl induced marginal changes in the pasting profiles of F100, F50, and F0. As the NaCl concentration increased (4.46% and 8.92%), the pasting viscosities were influenced by the added NaCl. Li et al. [27] also found that 1% and 2% NaCl addition showed no significant changes in starch pasting properties of wheat flour.

Table 2 summarizes changes in the pasting properties of the wheat flour/starch samples with different NaCl contents. NaCl significantly increased the time to peak viscosity (TTPV) only at the highest concentration (8.92%) in F0 formulations, while no significant effect was observed in F100 and F50. Increased TTPV values indicated that NaCl could delay the time for the wheat flour/starch suspension to reach peak viscosity. NaCl at a high concentration might inhibit water from entering starch granules through electrostatic screening [28]. When NaCl reached 8.92%, the TTPV of F100, F50, and F0 increased by 0.07, 0.20, and 0.33 min, respectively, indicating that the gluten proteins might weaken the inhibitory effect of NaCl on starch swelling. The effect of NaCl on peak viscosity (PV) was dependent on formulation. For F100, PV increased significantly only at higher concentrations (4.46% and 8.92%), with no significant change at 0~2%. For F50, a significant increase was observed starting from 1% NaCl. For F0, a significant increase was only observed at the highest concentration (8.92%) (Table 2). PV values reflected the degree of swelling in starch granules during RVA heating. The results indicated that NaCl increased the ability of wheat starch granules to absorb water and swell before their disruption [15]. This is consistent with previous literature reports, where Zheng et al. (2022) found that NaCl (50–200 mM) increased the PV of wheat starch from 1815 cP to 2043 cP [16]. The interaction between salt ions and starch hydroxyl groups weakened the fluidity of starch paste, and the strong interaction between NaCl (with higher concentrations) and water molecules decreased the fluidity of both water and starch molecules, leading to an increase in pasting viscosity [16]. Additionally, the addition of 8.92% NaCl increased the PV of F100, F50, and F0 by 19.43%, 16.68%, and 5.85%, respectively. The result was probably due to the interactions between NaCl and gluten proteins, which further increased the PV values. NaCl altered the trough viscosities (TVs) of the wheat flour/starch suspensions and consequently showed a significant effect on breakdown (BD) viscosity. BD represented the shear resistance and heat stability of swollen starch granules. As the amount of NaCl added increased (0% → 2% → 8.92%), the BD of F100 and F50 remained statistically unchanged at lower NaCl concentrations (0–2%) but increased significantly at higher levels (4.46% and 8.92%) (*p* < 0.05), while the BD of F0 showed a trend of first increasing and then decreasing. This indicated that NaCl at low concentrations (1% and 2%) promoted the disintegration of swollen starch granules in RVA, while at its high concentrations (4.46% and 8.92%), it still promoted the disintegration of swollen starch granules in samples with gluten proteins (F100 and F50) but exhibited inhibitory effects on the starch granule disintegration in samples without gluten proteins (F0). Similarly, Zheng et al. (2022) also found that the BD value of wheat starch decreased from 966 cP to 756 cP after NaCl (200 mM) was added [16]. Final viscosity (FV) represented the equilibrium viscosity of cooked starch after holding the paste at 50 °C, reflecting the cooked starch’s resistance to shear deformation and its gel-forming capacity [15]. FV of F100, F50, and F0 showed irregular change as NaCl was added. In particular, the FV values initially decreased upon the addition of 1–2% NaCl but subsequently increased at higher NaCl concentrations. Setback (SB) viscosity represented the tendency of short-term retrogradation of cooked starch. With the addition of NaCl, the SB value changes had different trends among F100, F50, and F0. Specifically, NaCl decreased SB of F0 at higher concentrations (4.46% and 8.92%). However, higher NaCl concentrations (4.46% and 8.92%) led to an increase in SB of F100 and F50. Interestingly, the addition of 1–2% NaCl decreased SB of F100 and F50, showing a retarding effect on the short-term retrogradation in these mianpi samples. Probably, Na^+^ and Cl^−^ interacted with starch chains to generate a Donnan potential, by which repulsion between starch chains was enhanced and intermolecular reassociation of starch chains was inhibited [29]. However, at high NaCl concentrations (4.46% and 8.92%), the presence of gluten proteins might counteract the inhibitory effect of NaCl on short-term starch retrogradation and instead facilitate the polymerization of starch molecular chains. Meanwhile, differences in FV and SB among the formulations (F100, F50, and F0) at the same NaCl concentration highlight the role of starch–protein composition in gelation and retrogradation. At 0% NaCl, FV increased in the order F0 > F50 > F100, and F0 provides the highest concentration of amylose and amylopectin for leaching and reassociation, thereby forming a denser gel network upon cooling [11]. In contrast, F100 contains non-starch components (mainly gluten and lipids), which dilute the starch concentration and hinder network formation. SB followed the order F0 > F50 > F100. SB reflects the retrogradation tendency of starch, where amylose molecules easily reassociate in the absence of interfering components. The presence of gluten proteins in F100 and F50 reduces retrogradation by physically impeding starch alignment and competing for water, thereby lowering SB. This inhibitory role of gluten is consistent with earlier reports [30].

### 3.3. Effects of NaCl on Qualities of Mianpi

#### 3.3.1. Color

Color serves as a critical sensory attribute in mianpi quality assessment, directly influencing consumers’ initial perception of product quality. Table 3 shows the L*, a*, and b* values of mianpi samples (F100, F50, and F0) with different NaCl concentrations. As shown in Table 3, F100 showed no significant change in L* and a* values by adding NaCl (*p* > 0.05), but NaCl addition (1% and 2%, *w*/*w*) significantly reduced b* value (*p* < 0.05). The decrease in b* value indicated less yellowness of the product [31]. In F50, NaCl decreased the L* and b* values but increased the a* value. The decrease in L* value of mianpi with NaCl indicated less light reflection or more opaqueness of the product [32]. The increase in a* value indicated more redness of the product. In F0, NaCl decreased the L* value (*p* < 0.05) but showed an insignificant effect on the a* and b* values (*p* > 0.05). The decrease in L* value of F0 indicated a darker color in the presence of NaCl. Similarly, Torres et al. [32] stated that the presence of NaCl (0.5–2.0%, *w*/*w*) decreased the L* value of gels from chestnut and rice flours while maintaining the a* and b* values. This phenomenon may be related to the characteristics of NaCl as a strong electrolyte. The sodium ions dissociated from NaCl reduce the water activity of the slurry, making it difficult for some starch granules to swell and disintegrate, leading to the existence of not fully gelatinized starch granules in the gel of mianpi, which leads to the decrease in light transmittance and lightness. Beyond the effect of NaCl, the mianpi formulations demonstrated distinct color properties. In the absence of NaCl, F100 and F50 exhibited significantly elevated L* and a* values compared to F0, with F100 additionally showing higher b* than both F50 and F0. The distinct chromatic profiles observed in F100 and F50 formulations originated from the modifying effects of gluten proteins on starch gelation, which probably operated through the synergistic mechanisms, i.e., protein–starch interactions altered the gelation kinetics, extending the network formation period, resulting in a denser and more uniform gel matrix compared to F0. In comparison with the control (without NaCl addition), the Δ*E* values of the samples with NaCl addition ranged from 1.09 to 3.30 (Table 3). According to literature reports, the color difference between products with Δ*E* < 3.7 cannot be observed by the human eye [31], so the color difference of mianpi samples caused by NaCl addition was not significant under human observation.

#### 3.3.2. Textural Properties

Mianpi is consumed in its cool state and texture is one of the major concerns for mianpi consumers. Table 4 shows the texture characteristics of mianpi samples (F100, F50, and F0) with different NaCl concentrations. For F100, whether NaCl was added or not, no significant changes in adhesion, elasticity, and chewiness were observed. Only when the NaCl addition was increased to 2% (*w*/*w*) did the hardness of F100 mianpi significantly decrease (*p* < 0.05). Fan et al. [7] found that NaCl had no obvious effects on the hardness, springiness, and chewiness of wheat noodles. For F50, the addition of NaCl increased the hardness, adhesiveness, and chewiness of mianpi. However, for F0, the hardness, adhesion, and chewiness of mianpi showed a decreasing trend with the increase in NaCl addition (0–2%). The decrease in hardness and chewiness may be attributed to the modification of the starch gel network by NaCl, potentially through its influence on water distribution and molecular interactions, e.g., by competing for water molecules and interfering with starch chain associations [12]. Similarly, Hedayati et al. [15] reported that the added NaCl decreased the texture parameters (hardness, cohesiveness, springiness, gumminess) of the gels of granular cold water swelling maize starches. However, as the NaCl addition amount increased, F50 showed an opposite trend in hardness and chewiness. This was probably associated with the role of Na^+^ ions in enhancing interactions between starch molecules and gluten protein components which helped the formation of a more rigid or denser network [33].

#### 3.3.3. Tensile Properties

Prior to consuming, mianpi is cut into strips, so tensile properties are considered as a key characteristic for the product. The tensile test involves stretching the sample to measure its extensibility and tensile strength, quantified by the force required to stretch the sample (tensile force, g) and the distance it can be stretched (extension, mm). As shown in Table 5, as the addition amount of NaCl increased, the tensile force values of F100 and F50 mianpi samples increased significantly (*p* < 0.05), while NaCl showed no obvious effect on the tensile force values of F0 mianpi samples (*p* > 0.05). The enhanced tensile strength observed in both F100 and F50 samples demonstrates that the incorporation of NaCl significantly reinforced the mianpi gel network structure. Moreover, the added NaCl increased the extension value of F100 mianpi samples while it had an insignificant effect on the extension value of F50 and F0 mianpi samples. The results implied that NaCl had an insignificant effect on the tensile properties of gels of extracted wheat starch (F0). Interestingly, Sangpring et al. [19] found that NaCl (3 and 5 g NaCl/100 g rice flour) decreased the tensile strength and increased the extensibility of cooked rice noodles and they explained that the decrease in the tensile strength of cooked rice noodles with NaCl was probably due to the inhibition of starch retrogradation by NaCl. However, Fan et al. [7] revealed that NaCl supplementation (5–50 g/kg) enhanced the tensile strength (force) of noodles prepared from Aikang 58 wheat flour, whereas it elicited inconsistent effects on noodles derived from two other wheat varieties. Chen et al. [34] indicated that the rupture force of hard wheat flour dough increased with the addition of NaCl and the rupture force (28.0 g) attained its maximum value when the dough contained 2.4% NaCl. These results implied that the role played by NaCl was influenced by the components in the products (such as starch and proteins).

### 3.4. Effects of NaCl on Viscoelastic Properties of Mianpi

**Figure 4:**
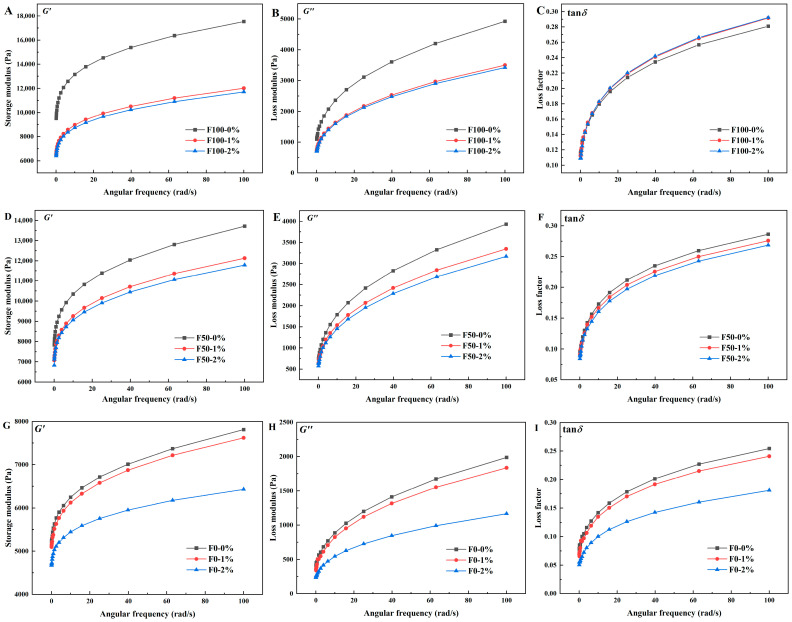
Frequency sweep profiles of mianpi with different levels of NaCl. (**A**) Storage modulus (*G*′) of F100; (**B**) Loss modulus (*G*″) of F100; (**C**) Loss factor (tan *δ*) of F100; (**D**) Storage modulus (*G*′) of F50; (**E**) Loss modulus (*G*″) of F50; (**F**) Loss factor (tan*δ*) of F50; (**G**) Storage modulus (*G*′) of F0; (**H**) Loss modulus (*G*″) of F0; (**I**) Loss factor (tan*δ*) of F0. All subfigures show data as a function of angular frequency.

The storage modulus (*G*′), loss modulus (*G*″), and loss factor (tan *δ* = *G*″/*G*′) of the mianpi samples were determined by sweeping the oscillation frequency. *G*′ and *G*″ represented the elasticity energy of the gel and the energy lost by the gel to resist shear, respectively [11]. As shown in Figure 4A,B,D,E,G,H, both *G*′ and *G*″ for all mianpi samples increased with rising frequency, demonstrating clear frequency-dependent behavior. Within the angular frequency range of 0–100 rad/s, *G*′ consistently surpasses *G*″, indicating that the samples displayed elastic gel-like structure [35]. Furthermore, the mianpi samples behaved as an elastic solid due to tan *δ* < 1 (Figure 4C,F,I). Figure 4 also demonstrates a dose-dependent reduction in viscoelastic properties: F100/F50 samples exhibited significant decreases in both *G*′ and *G*″ values at 1% NaCl, with further attenuation at 2% NaCl, while F0 samples showed comparable rheological weakening only at the higher NaCl concentration (2%). Previously, Zheng et al. [16] observed that NaCl concentration affected wheat starch gel rheology differently: elevated levels (150–200 mM) reduced *G*′ and *G*″, whereas lower concentrations (50–100 mM) increased both moduli. They explained that NaCl, at lower concentrations, promoted amylose chain polymerization via hydrogen bonding, strengthening the gel network structure. In the present study, the following explanation could be given for the reduction of *G*′ and *G*″: (1) sodium ions interacted with the hydroxyl groups in the starch molecules, hindering the polymerization of the starch molecules [16]. Meanwhile, (2) the Donnan potential induced by chloride ions surrounding starch molecules strengthened electrostatic repulsion between starch chains. This effect disrupted tight molecular packing and suppresses starch retrogradation, ultimately leading to reductions in both *G*′ and *G*” [36]. To some extent, *G*′ reflected the elastic quality of mianpi samples which referred to short-term retrogradation of starch [11]. The decrease in *G*′ agreed with the decreased setback viscosity from Section 3.2 RVA results. As shown in Figure 4C,F,I, increasing NaCl concentration led to divergent trends in value of tan *δ* across formulations. Specifically, F100 exhibited an elevated tan *δ*, suggesting reduced elasticity, whereas F50 and F0 displayed a decrease in tan *δ*, indicating enhanced elastic behavior. Consequently, in this study, gluten proteins governed the structural integrity of mianpi gels and mediated NaCl-induced alterations in their viscoelastic properties.

### 3.5. Effects of NaCl on Structural Characteristics of Starch Components in Mianpi

**Figure 5:**
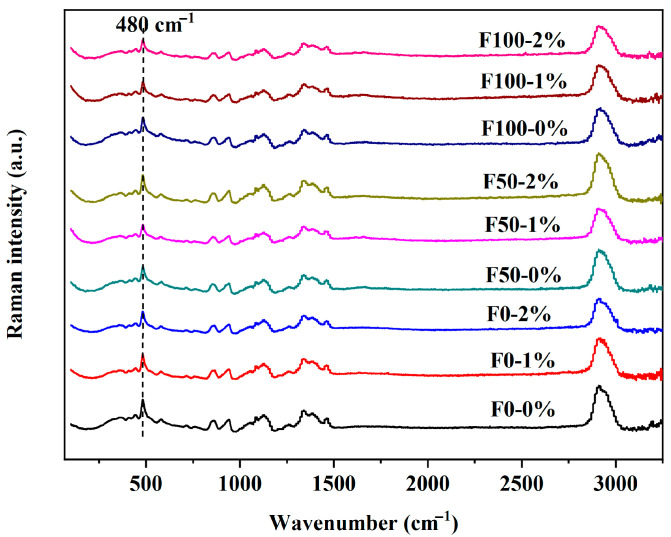
Raman spectra of mianpi with different levels of NaCl.

The Raman spectra of the mianpi samples are shown in Figure 5. The bands of F100, F50, and F0 mianpi samples with/without NaCl were similar, indicating that the characteristic band positions did not change after the addition of NaCl. As indicated by various studies, the characteristic band at 480 cm^−1^ in the Raman spectrum, originated from the glucose pyranose ring structure of starch, was a good indicator of the extent of the short-range molecular order of the double helices in starch [20]. According to Table 6, there was no significant change in the FWHM values of F100, F50, and F0 mianpi samples after adding NaCl (*p* > 0.05). Bilal et al. [37] concluded that the FWHM of Raman bands served as a reliable parameter for quantifying short-range molecular order in crystalline starch, while demonstrating limited applicability to amorphous starch domains. For the mianpi samples, amorphous starch dominated in the gel structure, so that NaCl-induced short-range molecular order change was probably not reflected in the FWHM of the Raman band at 480 cm^−1^.

**Figure 6:**
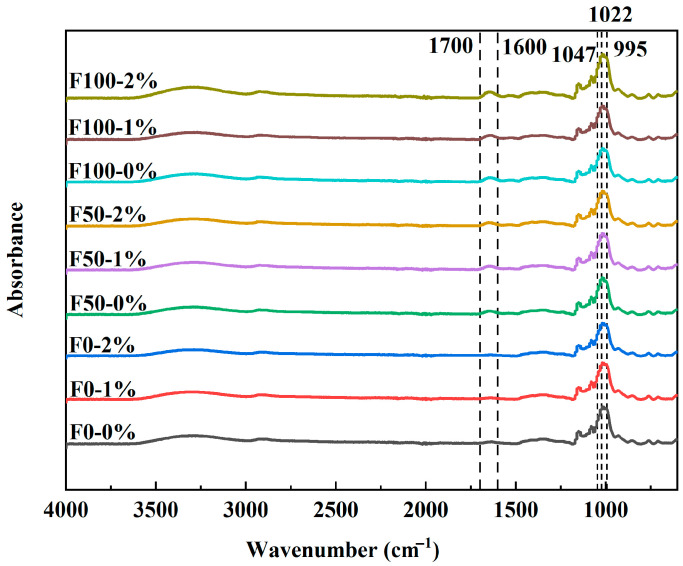
FTIR spectra of mianpi with different levels of NaCl.

As shown in Figure 6, the characteristic band positions of mianpi samples in the FTIR spectra were the same, indicating that the addition of NaCl did not cause the formation of new functional groups in the samples. According to published reports [38,39], the FTIR spectrum in the 800–1200 cm^−1^ range showed pronounced sensitivity to the conformational features of starch molecules. Notably, the bands at 1047 cm^−1^ and 995 cm^−1^ corresponded to the ordered crystalline domains of starch polymers, while the band at 1022 cm^−1^ originated from vibrational modes characteristic of amorphous starch regions. The absorbance ratio *R*_1047/1022_ (1047/1022 cm^−1^) served as a quantitative indicator of the degree of order in starch, whereas *R*_1022/995_ (1022/995 cm^−1^) reflected the relative proportion of amorphous to ordered structures within starch polymers. According to Table 6, as the addition amount of NaCl increased from 0% to 2%, a decrease in *R*_1047/1022_ values was found in F100 and F0 samples, whereas an increment of *R*_1047/1022_ values was found in F50 samples. Meanwhile, the added NaCl led to an increase in the *R*_1022/995_ values of F100 and F0 samples while it showed insignificant effect on the *R*_1022/995_ values of F50 samples. In addition, when comparing mianpi samples without NaCl, it was observed that F0 showed the greatest *R*_1047/1022_ value and the lowest *R*_1022/995_ value. It indicated that the coexisting components in wheat flours (mainly gluten proteins) hindered the formation of short-range order structure in the starch gels.

### 3.6. Effects of NaCl on Structural Characteristics of Proteins in Mianpi

The band region from 1600–1700 cm^−1^ (amide I) of the FTIR spectrum was sensitive to protein conformational changes [21]. This region included information related to the secondary structure of proteins (*α*-helix, *β*-sheet, *β*-turn, and random coil) [40]. The bands in the amide I region are generally assigned as: *α*-helix (1650–1660 cm^−1^), *β*-sheet (1610–1640 cm^−1^ and 1670–1690 cm^−1^), *β*-turn (1660–1670 cm^−1^ and 1690–1700 cm^−1^), and random coil (1640–1650 cm^−1^) [34,41]. The relative contents of protein secondary structures could be calculated using PeakFit software by deconvolution of the amide I band (1600–1700 cm^−1^) including baseline correction and second-order derivative fitting. Secondary structure proportions of proteins in different mianpi samples were calculated and are listed in Table 7. F0 was made from extracted wheat flours so that the protein secondary structure in F0 was not calculated. According to Table 7, all the samples had a higher proportion of *β*-sheets than *α*-helices, *β*-turns, and random coils, indicating that the *β*-sheet was the dominant secondary structure in the mianpi proteins. However, a significant change in secondary structure proportions (*α*-helix, *β*-sheet, *β*-turn, and random coil) in the presence of NaCl was not found. The result was consistent with the study by Gui and Lu [41], who found that different NaCl concentrations (0.6–1.76%) had no significant effect on the secondary structures (*β*-sheet, random coil, *α*-helix, and *β*-turn) of gluten proteins in cooked noodles.

## 4. Conclusions

NaCl serves as a critical modifier in wheat flour–starch systems, affecting rheology, texture, and molecular structure. Adding NaCl could improve processing stability and mianpi quality, particularly in retarding retrogradation and enhancing tensile strength. In particular, NaCl (1–2%) decreased the mianpi slurry’s apparent viscosity. NaCl demonstrated concentration-dependent effects on pasting properties. With increasing NaCl concentration, peak viscosity exhibited a proportional rise, while breakdown values diverged—F100/F50 showed sustained increases whereas F0 demonstrated a unimodal response (initial rise followed by decline). Minimum final viscosity was achieved at 1–2% NaCl. Notably, 1–2% NaCl reduced setback in F100 and F50, indicating a retarding effect on short-term retrogradation in these mianpi samples. Statistically significant lightness reduction (*p* < 0.05) was detected in F50 and F0 formulations by NaCl addition, though these changes remained imperceptible to human observers. NaCl induced irregular variations in hardness, adhesiveness, springiness, and chewiness in mianpi formulations F50 and F0, while the texture of mianpi F100 stayed mostly the same, except 2% NaCl made it significantly softer. While F0 remained unaffected, NaCl consistently improved tensile strength in F100 and F50. In all mianpi formulations, increasing NaCl concentrations induced a progressive suppression of storage (*G’*) and loss (*G*″) moduli of the mianpi samples. FTIR analysis revealed that NaCl decreased the short-range order of starch in F100 and F0 but enhanced it in F50. However, this molecular reorganization was not reflected in the Raman 480 cm^−1^ band full width at half maximum. NaCl had no significant effect on the secondary structure of gluten proteins in mianpi. These findings provide valuable insights for the manufacturers developing starch-gel-based foods containing NaCl, flour, and starches.

## Figures and Tables

**Table 1:** The fitting parameters of Power-Law model obtained from the shear stress versus shear rate curves of the slurries with different levels of NaCl.

Samples	*K* (Pa·s*ⁿ*)	*n*	*R* ^2^
F50-0%	0.29 ± 0.03 ^d^	0.82 ± 0.01 ^a^	1
F50-1%	0.28 ± 0.01 ^d^	0.80 ± 0.00 ^a^	1
F50-2%	0.27 ± 0.02 ^d^	0.82 ± 0.01 ^a^	1
F100-0%	2.27 ± 0.09 ^b^	0.70 ± 0.00 ^b^	0.997
F100-1%	2.96 ± 0.11 ^a^	0.60 ± 0.01 ^c^	0.999
F100-2%	2.00 ± 0.11 ^c^	0.69 ± 0.01 ^b^	0.998

Data bearing different lowercase letters in the same column are significantly different (*p* < 0.05). Abbreviations: *K*, the consistency coefficient; *n*, the flow behavior index; *R*^2^, the correlation coefficients.

**Table 2:** Pasting parameters of wheat flour (F100), wheat flour–starch mix (F50), and wheat starch (F0) with different levels of NaCl.

Samples	NaCl Content (% *w*/*w*)	TTPV (min)	PV (cP)	TV (cP)	BD (cP)	FV (cP)	SB (cP)
F100	0	5.40 ± 0.09 ^e^	2388 ± 30 ^f^	1412 ± 20 ^f^	976 ± 10 ^g^	2547 ± 21 ^h^	1135 ± 1 ^g^
	1	5.40 ± 0.00 ^e^	2349 ± 68 ^f^	1321 ± 10 ^f^	1028 ± 78 ^g^	2351 ± 11 ^i^	1030 ± 21 ^h^
	2	5.40 ± 0.00 ^e^	2426 ± 8 ^f^	1321 ± 3 ^f^	1105 ± 6 ^fg^	2377 ± 11 ^i^	1056 ± 14 ^gh^
	4.46	5.43 ± 0.05 ^e^	2744 ± 60 ^e^	1382 ± 17 ^f^	1362 ± 77 ^de^	2636 ± 23 ^h^	1254 ± 40 ^f^
	8.92	5.47 ± 0.09 ^e^	2852 ± 42 ^de^	1393 ± 25 ^f^	1459 ± 16 ^bcd^	2763 ± 65 ^g^	1370 ± 40 ^e^
F50	0	5.43 ± 0.05 ^e^	2991 ± 55 ^d^	1768 ± 30 ^e^	1223 ± 25 ^ef^	3300 ± 42 ^ef^	1532 ± 12 ^d^
	1	5.50 ± 0.05 ^de^	3141 ± 23 ^c^	1750 ± 8 ^e^	1391 ± 15 ^cd^	3217 ± 24 ^f^	1467 ± 16 ^e^
	2	5.53 ± 0.00 ^de^	3230 ± 30 ^c^	1751 ± 11 ^e^	1479 ± 19 ^bcd^	3216 ± 34 ^f^	1465 ± 23 ^e^
	4.46	5.63 ± 0.05 ^cde^	3458 ± 49 ^ab^	1847 ± 2 ^e^	1612 ± 47 ^ab^	3374 ± 15 ^de^	1527 ± 13 ^d^
	8.92	5.63 ± 0.05 ^cde^	3490 ± 67 ^ab^	1843 ± 24 ^e^	1647 ± 91 ^a^	3424 ± 32 ^d^	1581 ± 8 ^c^
F0	0	5.80 ± 0.00 ^bc^	3418 ± 6 ^b^	2061 ± 6 ^cd^	1357 ± 0 ^de^	4072 ± 31 ^b^	2011 ± 25 ^a^
	1	5.87 ± 0.19 ^bc^	3526 ± 42 ^ab^	2111 ± 93 ^c^	1415 ± 52 ^cd^	4107 ± 43 ^b^	1996 ± 50 ^a^
	2	5.73 ± 0.00 ^cd^	3513 ± 3 ^ab^	1965 ± 32 ^d^	1549 ± 29 ^abc^	3968 ± 30 ^c^	2004 ± 2 ^a^
	4.46	5.97 ± 0.05 ^ab^	3566 ± 53 ^ab^	2214 ± 49 ^b^	1352 ± 4 ^de^	4152 ± 11 ^b^	1938 ± 60 ^b^
	8.92	6.13 ± 0.00 ^a^	3618 ± 68 ^a^	2427 ± 0 ^a^	1191 ± 68 ^f^	4312 ± 25 ^a^	1885 ± 25 ^b^

Values are mean ± standard deviation (*n* = 3). Data bearing different lowercase letters in the same column are significantly different (*p* < 0.05) as determined by one-way ANOVA and Duncan’s multiple range test. Abbreviations: TTPV, time to peak viscosity; PV, peak viscosity; TV, trough viscosity; BD, breakdown; FV, final viscosity; SB, setback.

**Table 3:** The color properties of mianpi with different levels of NaCl.

Samples	NaCl Content (% *w*/*w*)	L*	a*	b*	Δ*E*
F100	0	58.13 ± 1.17 ^ab^	−1.90 ± 0.22 ^d^	3.38 ± 0.40 ^a^	NA
	1	59.24 ± 0.42 ^a^	−1.94 ± 0.09 ^d^	1.74 ± 0.17 ^b^	2.02 ± 0.15
	2	58.26 ± 0.59 ^a^	−2.03 ± 0.19 ^d^	1.54 ± 0.18 ^b^	1.95 ± 0.16
F50	0	56.89 ± 0.76 ^bc^	−1.75 ± 0.11 ^cd^	−1.65 ± 0.23 ^c^	NA
	1	56.26 ± 0.67 ^c^	−1.53 ± 0.16 ^c^	−2.28 ± 0.36 ^cd^	1.09 ± 0.51
	2	54.97 ± 0.41 ^d^	−1.55 ± 0.14 ^c^	−2.51 ± 0.39 ^d^	2.14 ± 0.49
F0	0	53.17 ± 0.69 ^e^	1.11 ± 0.14 ^ab^	−2.01 ± 0.32 ^cd^	NA
	1	51.35 ± 1.13 ^f^	0.84 ± 0.26 ^b^	−2.08 ± 0.43 ^cd^	2.89 ± 0.57
	2	51.08 ± 0.50 ^f^	1.27 ± 0.23 ^a^	−2.28 ± 0.73 ^cd^	3.30 ± 0.25

Data bearing different lowercase letters in the same column are significantly different (*p* < 0.05). Abbreviations: L*, lightness; a*, level of redness or greenness; b*, level of yellowness or blueness; Δ*E*, color difference.

**Table 4:** The texture properties of mianpi with different levels of NaCl.

Samples	NaCl Content (% *w*/*w*)	Hardness (g)	Adhesiveness (g·s)	Springiness (-)	Chewiness (mJ)
F100	0	5707.70 ± 341.43 ^b^	−235.74 ± 21.08 ^c^	0.908 ± 0.013 ^a^	3743.26 ± 170.28 ^a^
	1	5713.03 ± 194.41 ^b^	−256.40 ± 36.65 ^c^	0.921 ± 0.034 ^a^	3740.06 ± 169.10 ^a^
	2	5279.06 ± 253.82 ^cd^	−241.68 ± 28.37 ^c^	0.905 ± 0.006 ^a^	3525.74 ± 157.79 ^ab^
F50	0	4709.56 ± 193.74 ^e^	−220.65 ± 34.32 ^c^	0.917 ± 0.021 ^a^	3139.95 ± 325.46 ^b^
	1	5014.91 ± 252.63 ^d^	−271.52 ± 7.80 ^d^	0.868 ± 0.036 ^b^	3279.42 ± 241.09 ^b^
	2	5351.41 ± 107.72 ^cd^	−344.52 ± 21.32 ^e^	0.895 ± 0.024 ^a^	3692.62 ± 174.44 ^ab^
F0	0	6392.36 ± 471.53 ^a^	−91.04 ± 23.62 ^b^	0.677 ± 0.085 ^d^	3537.98 ± 391.21 ^ab^
	1	5721.05 ± 207.45 ^b^	−66.51 ± 5.81 ^a^	0.762 ± 0.053 ^c^	3431.04 ± 272.58 ^ab^
	2	4736.76 ± 378.25 ^e^	−48.04 ± 9.32 ^a^	0.600 ± 0.031 ^d^	2038.85 ± 166.68 ^c^

Data bearing different lowercase letters in the same column are significantly different (*p* < 0.05).

**Table 5:** The tensile properties of mianpi with different levels of NaCl.

Samples	NaCl Content (% *w*/*w*)	Tensile Force (g)	Extension (mm)
F100	0	91.49 ± 6.53 ^c^	−11.78 ± 0.52 ^b^
	1	100.53 ± 6.39 ^ab^	−12.39 ± 0.34 ^b^
	2	101.64 ± 6.50 ^ab^	−13.04 ± 0.25 ^b^
F50	0	96.95 ± 7.71 ^bc^	−11.90 ± 0.50 ^b^
	1	118.17 ± 9.98 ^a^	−12.13 ± 0.58 ^b^
	2	120.06 ± 8.63 ^a^	−12.49 ± 0.46 ^b^
F0	0	102.54 ± 16.76 ^ab^	−7.97 ± 1.25 ^a^
	1	98.57 ± 9.22 ^bc^	−8.01 ± 0.73 ^a^
	2	101.87 ± 5.37 ^ab^	−8.63 ± 0.42 ^a^

Data bearing different lowercase letters in the same column are significantly different (*p* < 0.05).

**Table 6:** The short-range order structure of starch in mianpi with different levels of NaCl.

Samples	NaCl Content (% *w*/*w*)	FWHM	*R*_1047/1022_	*R*_1022/995_
F100	0	19.54 ± 0.51 ^a^	0.585 ± 0.002 ^e^	0.842 ± 0.008 ^abc^
	1	19.51 ± 0.02 ^a^	0.559 ± 0.009 ^f^	0.873 ± 0.016 ^ab^
	2	19.44 ± 0.22 ^a^	0.558 ± 0.002 ^f^	0.888 ± 0.071 ^ab^
F50	0	19.48 ± 0.82 ^a^	0.659 ± 0.008 ^c^	0.899 ± 0.018 ^a^
	1	19.18 ± 0.13 ^a^	0.679 ± 0.004 ^b^	0.892 ± 0.002 ^ab^
	2	18.77 ± 0.62 ^a^	0.681 ± 0.008 ^ab^	0.886 ± 0.006 ^ab^
F0	0	19.23 ± 0.00 ^a^	0.693 ± 0.002 ^a^	0.760 ± 0.005 ^d^
	1	19.44 ± 1.03 ^a^	0.603 ± 0.012 ^d^	0.773 ± 0.014 ^cd^
	2	18.70 ± 0.78 ^a^	0.584 ± 0.006 ^e^	0.811 ± 0.002 ^bcd^

Data bearing different lowercase letters in the same column are significantly different (*p* < 0.05). Abbreviations: FWHM, full width at half maximum; *R*_1047/1022_, the absorbance ratio at 1047 cm^−1^ and 1022 cm^−1^; *R*_1022/995,_ the absorbance ratio at 1022 cm^−1^ and 995 cm^−1^.

**Table 7:** The protein secondary structure in mianpi with different levels of NaCl.

Samples	NaCl Content (% *w*/*w*)	*β*-Sheet	Random Coil	*α*-Helix	*β*-Turn
F100	0	40.95 ± 0.44 ^a^	21.94 ± 0.56 ^a^	18.63 ± 0.66 ^a^	18.48 ± 0.59 ^a^
	1	39.32 ± 0.32 ^a^	21.04 ± 0.08 ^a^	19.06 ± 0.09 ^a^	20.57 ± 0.49 ^a^
	2	39.23 ± 1.38 ^a^	20.99 ± 1.50 ^a^	19.36 ± 0.93 ^a^	20.42 ± 1.53 ^a^
F50	0	38.92 ± 0.56 ^a^	21.77 ± 0.76 ^a^	19.08 ± 0.54 ^a^	20.24 ± 0.91 ^a^
	1	39.01 ± 0.87 ^a^	21.79 ± 2.27 ^a^	18.17 ± 0.74 ^a^	21.02 ± 1.95 ^a^
	2	38.41 ± 0.04 ^a^	22.62 ± 0.14 ^a^	18.82 ± 0.57 ^a^	20.15 ± 0.70 ^a^
F0	0	NA	NA	NA	NA
	1	NA	NA	NA	NA
	2	NA	NA	NA	NA

Data bearing different lowercase letters in the same column are significantly different (*p* < 0.05).

## Data Availability

The original contributions presented in this study are included in the article. Further inquiries can be directed to the corresponding authors.

## References

[1] Albarracín W., Sánchez I.C., Grau R., Barat J.M. (2011). Salt in food processing; usage and reduction: A review. Int. J. Food Sci. Technol..

[2] Sun X., Koksel F., Scanlon M.G., Nickerson M.T., Arntfield S.D. (2022). Effects of water, salt, and mixing on the rheological properties of bread dough at large and small deformations: A review. Cereal Chem..

[3] Avramenko N.A., Tyler R.T., Scanlon M.G., Hucl P., Nickerson M.T. (2018). The chemistry of bread making: The role of salt to ensure optimal functionality of its constituents. Food Rev. Int..

[4] Silow C., Axel C., Zannini E., Arendt E.K. (2016). Current status of salt reduction in bread and bakery products—A review. J. Cereal Sci..

[5] Belz M.C.E., Axel C., Beauchamp J., Zannini E., Arendt E.K., Czerny M. (2017). Sodium chloride and its influence on the aroma profile of yeasted bread. Foods.

[6] Carcea M., Narducci V., Turfani V., Mellara F. (2020). A comprehensive study on the influence of sodium chloride on the technological quality parameters of soft wheat dough. Foods.

[7] Fan H., Fu F., Chen Y., Liu M., Ai Z., Bian K. (2020). Effect of NaCl on rheological properties of dough and noodle quality. J. Cereal Sci..

[8] Wang J.-R., Guo X.-N., Xing J.-J., Zhu K.-X. (2020). Revealing the effect mechanism of NaCl on the rheological properties of dough of Chinese traditional hand-stretched dried noodles. Food Chem..

[9] Zhao G., Liu C., Li L., Li J., Wang J., Fan X., Zheng X. (2024). Structural characteristics and paste properties of wheat starch in natural fermentation during traditional Chinese Mianpi processing. Int. J. Biol. Macromol..

[10] Shang J., Liu C., Li L., Hong J., Liu M., Liu Z., Zhao B., Zheng X. (2024). Effect of salt and alkali on the viscoelastic behavior of noodle dough sheet with different wheat starch granule sizes. Food Res. Int..

[11] Li E., Lv J., Huo D., Jia B., Li C. (2023). Importance of amylose chain-length distribution in determining starch gelatinization and retrogradation property of wheat flour in the presence of different salts. Carbohydr. Polym..

[12] Chen C., Li G., Corke H., Zhu F. (2023). Physicochemical properties of starch in sodium chloride solutions and sucrose solutions: Importance of starch structure. Food Chem..

[13] Nicol T.W.J., Isobe N., Clark J.H., Matubayasi N., Shimizu S. (2019). The mechanism of salt effects on starch gelatinization from a statistical thermodynamic perspective. Food Hydrocoll..

[14] Chen Z., Li X., Tong Z., Huang J., Pu H., Zhong H. (2023). The effects of NaCl on starch molecular conformation. Starch-Stärke.

[15] Hedayati S., Shahidi F., Majzoobi M., Koocheki A., Farahnaky A. (2020). Structural, rheological, pasting and textural properties of granular cold water swelling maize starch: Effect of NaCl and CaCl_2_. Carbohydr. Polym..

[16] Zheng L., Ren A., Liu R., Xing Y., Yu X., Jiang H. (2022). Effect of sodium chloride solution on quality of 3D-printed samples molded using wheat starch gel. Food Hydrocoll..

[17] (2010). General Pasting Method for Wheat or Rye Flour or Starch—Using the Rapid Visco Analyzer.

[18] Xiao X., Yang L., Xu Z., Huang P., Shu C., Song S., Zhang Y., Pei H. (2024). Research on rice starch gel preparation and crosslink network structure–rheological property based on direct-writing 3D printing. Heliyon.

[19] Chen G., Ehmke L., Sharma C., Miller R., Faa P., Smith G., Li Y. (2019). Physicochemical properties and gluten structures of hard wheat flour doughs as affected by salt. Food Chem..

[20] Liu X., Luan H., Yu J., Wang S., Wang S., Copeland L. (2020). A method for characterizing short-range molecular order in amorphous starch. Carbohydr. Polym..

[21] Zhang J., Li X., Ren X., An Y., Song X., Zhao Y., Wen Y., Zhang W. (2024). Changes in physicochemical characteristics of wheat flour and quality of fresh wet noodles induced by microwave treatment. Grain Oil Sci. Technol..

[22] Wang Y., Ye F., Liu J., Zhou Y., Lei L., Zhao G. (2018). Rheological nature and dropping performance of sweet potato starch dough as influenced by the binder pastes. Food Hydrocoll..

[23] Mioduszewski Ł., Cieplak M. (2021). Viscoelastic properties of wheat gluten in a molecular dynamics study. PLoS Comput. Biol..

[24] Liu C., Zhang H., Chen R., Chen J., Liu X., Luo S., Chen T. (2021). Effects of creeping fig seed polysaccharide on pasting, rheological, textural properties and in vitro digestibility of potato starch. Food Hydrocoll..

[25] Saleh M. (2018). Wheat batter physical properties as influenced by starch/flour types and egg contents. J. Food Meas. Charact..

[26] Xue J., Ngadi M. (2006). Rheological properties of batter systems formulated using different flour combinations. J. Food Eng..

[27] Li M., Sun Q.-J., Han C.-W., Chen H.-H., Tang W.-T. (2018). Comparative study of the quality characteristics of fresh noodles with regular salt and alkali and the underlying mechanisms. Food Chem..

[28] Li D., Fayet C., Homer S. (2020). The effect of sodium chloride on gluten network formation and dough rheology in wheat flour. J. Cereal Sci..

[29] Tan H.-L., Tan T.-C., Easa A.M. (2020). The use of selected hydrocolloids and salt substitutes on structural integrity, texture, sensory properties, and shelf life of fresh no salt wheat noodles. Food Hydrocoll..

[30] Guo J., Lian X., Kang H., Gao K., Li L. (2016). Effects of glutenin in wheat gluten on retrogradation of wheat starch. Eur. Food Res. Technol..

[31] Bai J., Dong M., Li J., Tian L., Xiong D., Jia J., Yang L., Liu X., Duan X. (2022). Effects of egg white on physicochemical and functional characteristics of steamed cold noodles (a wheat starch gel food). LWT-Food Sci. Technol..

[32] Torres M.D., Raymundo A., Sousa I. (2014). Influence of Na^+^, K^+^ and Ca^2+^ on mechanical and structural properties of gels from chestnut and rice flours. Carbohydr. Polym..

[33] Sangpring Y., Fukuoka M., Ratanasumawong S. (2015). The effect of sodium chloride on microstructure, water migration, and texture of rice noodle. LWT-Food Sci. Technol..

[34] Tao H., Lu F., Zhu X.-F., Xu G.-X., Xie H.-Q., Xu X.-M., Wang H.-L. (2021). Removing surface proteins promote the retrogradation of wheat starch. Food Hydrocoll..

[35] Li W., Zhang K., Qin Y., Li M., Li H., Guo M., Xu T., Sun Q., Ji N., Xie F. (2025). Effects of sodium chloride on the textural attributes, rheological properties, microstructure, and 3D printing performance of rice starch-curdlan composite gel. Food Chem..

[36] Lu H., Tian Y., Ma R. (2023). Assessment of order of helical structures of retrograded starch by Raman spectroscopy. Food Hydrocoll..

[37] Bilal M., Zhang Y., Li D., Xie C., Yang R., Gu Z., Jiang D., Wang P. (2023). Optimizing techno-functionality of germinated whole wheat flour steamed bread via glucose oxidase (Gox) and pentosanase (Pn) enzyme innovation. Grain Oil Sci. Technol..

[38] Ma R., Tian Y., Chen L., Cai C., Jin Z. (2019). Effects of cooling rate on retrograded nucleation of different rice starch-aromatic molecule complexes. Food Chem..

[39] Fevzioglu M., Ozturk O.K., Hamaker B.R., Campanella O.H. (2020). Quantitative approach to study secondary structure of proteins by FT-IR spectroscopy, using a model wheat gluten system. Int. J. Biol. Macromol..

[40] Zhan J., Ma S., Wang X.-X., Li L., Zheng X.-L. (2019). Effect of baked wheat germ on gluten protein network in steamed bread dough. Int. J. Food Sci. Technol..

[41] Gui J., Lu Q. (2021). Effects of anions on water distribution, protein secondary structure and microstructure of noodles. J. Chin. Inst. Food Sci. Technol..

